# Retrospective Analysis of Postoperative Nonhepatic Outcomes Following Major Liver Resection

**DOI:** 10.7759/cureus.60311

**Published:** 2024-05-14

**Authors:** Ahmed Bilal Akhtar, Saad Ur Rehman, Shafiq Ur Rehman, Hassaan Bari

**Affiliations:** 1 Anesthesia and Critical Care, King Faisal Specialist Hospital and Research Centre, Riyadh, SAU; 2 Anesthesiology, Shaukat Khanum Memorial Cancer Hospital and Research Centre, Lahore, PAK; 3 Anesthesia, Heartlands Hospital, Birmingham, GBR; 4 Surgical Oncology, Shaukat Khanum Memorial Cancer Hospital and Research Centre, Lahore, PAK

**Keywords:** length of stay in the icu, output factors, perioperative outcomes, anesthesia, liver resection, morbidity and mortality, postoperative blood loss

## Abstract

Background

Liver surgery is a major and challenging procedure for the surgeon, the anesthetist, and the patient. The objective of this study was to evaluate the postoperative nonhepatic complications of patients undergoing liver resection surgery with perioperative factors.

Methods

We retrospectively analyzed 79 patients who underwent liver resection surgeries at the Shaukat Khanum Memorial Cancer Hospital and Research Centre in Lahore, Pakistan, from July 2015 to December 2022.

Results

The mean age at the time of surgery was 53 years (range: 3-77 years), and the mean BMI was 26.43 (range: 15.72-38.0 kg/m^2^). Of the total patients, 44.3 % (n = 35) had no comorbidities, 26.6% (n=21) had one comorbidity, and 29.1% (n=23) had two or more comorbidities. Patients in whom the blood loss was more than 375 ml required postoperative oxygen inhalation with a significant relative risk of 2.6 (p=0.0392) and an odds ratio of 3.5 (p=0.0327). Similarly, patients who had a surgery time of more than five hours stayed in the hospital for more than seven days, with a statistically significant relative risk of 2.7 (p=0.0003) and odds ratio of 7.64 (p=0.0001). The duration of surgery was also linked with the possibility of requiring respiratory support, with a relative risk of 5.0 (p=0.0134) and odds ratio of 5.73 (p=0.1190).

Conclusion

Patients in our cohort who had a prolonged duration of surgery received an increased amount of fluids, and a large volume of blood loss was associated with prolonged stay in the ICU (>2 days), hospital admission (>7 days), ICU readmission, and increased incidence of cardiorespiratory, neurological, and renal disturbances postoperatively.

## Introduction

Cancer is one of the most lethal noncommunicable diseases in humans [1]. The international communities have been trying to adapt measures to decrease the burden of disease but still, the numbers are very high, especially in the third world countries. Primary hepatic carcinoma is the fourth most common malignancy after lung, colorectal, and stomach cancers [2]. In 2017, approximately 950,000 cases of liver cancer with a mortality rate of 819,000 were reported worldwide [3]. Liver resection is the primary treatment for many different types of hepatic, pancreatic, and biliary diseases [4].

Liver surgery is a major and challenging procedure for the surgeon, anesthetist, and patient as well [5]. Major morbidity ranges from 17% in benign and 27% in malignant diseases, with a mortality risk of up to 5%. There is an increased risk of thromboembolic events of up to 50% [6]. Postoperative nausea and vomiting also occur in approximately 50% of patients. Perioperative stress response increases during major liver resection. The high incidence of postoperative complications and variable anesthetic practices have resulted in poor analgesic control, increased resource utilization, and possible adverse outcomes [7].

Postoperative pulmonary complications and intraoperative blood loss are essential determinants of postoperative outcomes after liver resection [8]. The use of a lung-protective mechanical ventilation strategy, moderate levels of positive end-expiratory pressure (PEEP), and repeated recruitment maneuvers significantly reduced postoperative pulmonary complications [9]. Unfortunately, it is also widely accepted that PEEP should not be used in liver resection because it can increase central and hepatic venous pressures, leading to higher blood loss [10]. Increased blood loss during liver resection is a risk factor for increased postoperative morbidity and mortality [11]. All of these factors affect the length of postoperative stay and cost-effectiveness.

Liver surgery was initiated at our institute in 2013. To date, no study has been conducted to determine postoperative nonhepatic outcomes following liver resection in our population. The rationale of this study was to determine the perioperative factors leading to increased morbidity and mortality following major liver resection, the correlation of postoperative complications such as respiratory issues, acute kidney injury, metabolic disturbances, length of stay and readmission in critical care, total length of hospital stay, cardiac events, and pulmonary embolism.

## Materials and methods

After obtaining approval from the institutional review board (IRB), the retrospective study was started. Data regarding liver resection surgeries for primary hepatic carcinoma from 2015 to 2022 were extracted from the Hospital Information System (HIS). The HIS of our institute records information in real time, and all the data are stored therein. Thus, it is very easy to obtain all the information at any time and allows for an accurate retrospective review of the data. Patients who underwent emergency surgery, American Society of Anesthesiologists (ASA) V, with Child Pugh's score of more than 6 or additional nonliver surgery were not included.

Data retrieved included demographics; preoperative evaluation regarding comorbid conditions such as hypertension, diabetes mellitus, ischemic heart disease, chronic kidney disease, and chronic liver disease; and the severity and postoperative outcome of the patient. Information regarding intraoperative events was also extracted, including duration of surgery, total fluid intake, blood loss during surgery, urine output, hemodynamic and metabolic profiles, electrolyte balance, lactate levels, and if the patient required any vasopressor support.

Postoperative data were acquired, including patient destination, hemodynamics, length of stay from the day of surgery until the day of discharge from the hospital, and stay in critical care, as if the patient was admitted only once during their stay in the hospital or were re-admitted for postoperative complications. Data for any postoperative complications, such as renal (confirmed by RIFLE criteria) or pulmonary (if treatment was received) complications, were also recorded. The postoperative lactate and pH levels were measured. The incidence of pulmonary embolism confirmed by computed tomography angiography was also included in the data. The overall incidences of morbidity and mortality were also calculated.

All statistical analyses were performed using IBM SPSS Statistics for Windows, Version 23 (Released 2015; IBM Corp., Armonk, New York, United States). For continuous variables, the mean and median values were calculated. Frequencies and percentages were calculated for categorical variables. Odds ratios and relative risks were calculated for intraoperative risk factors in relation to postoperative complications.

Operational definitions

Major Liver Resection

Major liver resection is defined as a resection of three or more Couinaud segments.

Length of Stay

Length of stay is the time from the day of surgery till the day of discharge from the hospital.

Acute Kidney Injury

Acute kidney injury is defined as per RIFLE criteria.

Respiratory Support

Respiratory support is ventilator requirement or continuous positive airway pressure or bilevel positive airway pressure.

## Results

**Figure 1:**
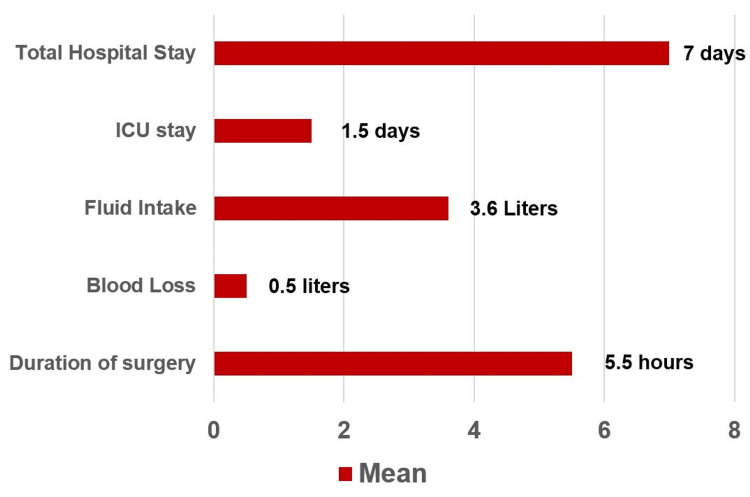
Mean values of various categories. ICU: Intensive care unit

After excluding the above-mentioned patients, 79 patients underwent liver resection between 2015 and 2022. All of these patients had Child-Pugh class A, having surgery as the first line treatment with curative intent. Table 1 shows the general characteristics of the patients enrolled in this study. Figure 1 shows the mean values of the important studied variables.

**Table 1:** Demographic variables. ASA: American Society of Anesthesiologists; IHD: ischemic heart disease; COPD: chronic obstructive pulmonary disease; CKD: chronic kidney disease; CLD: chronic liver disease

Variables		Values
Age (years) – Median		55
Age (years) - Category	Equal or below 40	13.9% (n=11)
	Above 40	86.1% (n=68)
Gender	Males	45.6% (n=36)
	Females	54.4% (n=43)
BMI (kg/m^2^)	Less than 25	41.8% (n=33)
	More than 25	58.2% (n=46)
Demographics	Afghanistan	5.1% (n=4)
	Azad Jammu and Kashmir	1.3% (n=1)
	Baluchistan	2.5% (n=2)
	KPK	21.5% (n=17)
	Punjab	69.6% (n=55)
Comorbidities	Hypertension	36.7% (n=29)
	Diabetes	29.1% (n=23)
	IHD	7.6% (n=6)
	COPD	3.8% (n=3)
	Asthma	2.5% (n=2)
	CKD	2.5% (n=2)
	CLD	10.1% (n=8)
ASA Grade	ASA I	3.8% (n=3)
	ASA II	67.1% (n=53)
	ASA III	21.5% (n=17)
	ASA IV	7.6 (n=6)
Addiction	Smoking	34.2% (n=26)
	Tobacco Chewing	6.3% (n=5)
	Alcoholism	7.6% (n=6)

Tables 2, 3 show the relative risk and odds ratio of intraoperative factors associated with postoperative outcomes. The p-values for the tables were calculated using online software MedCalc (MedCalc Software, Belgium) where the p-value is the area of the normal distribution that falls outside ± z [12]. The total population was divided into two groups according to the median values of all three major intraoperative factors. Statistically significant findings included increased hospital stay with surgery time > 5 h (relative risk: 2.7, odds ratio: 7.6), blood loss > 375ml (relative risk: 2.3, odds ratio: 5.9), and fluid intake > 3400ml (relative risk: 2.1, odds ratio: 1.0). There was requirement for postoperative oxygen inhalation when blood loss was > 375 ml (relative risk: 2.6, odds ratio: 3.5). There was increased risk of postoperative respiratory support when surgery time was > 5 h (relative risk 5.0, odds ratio 5.7).

**Table 2:** Relative risk of outcomes to intraoperative factors with p-values. p-value significant: <0.05 ICU: Intensive care unit

Outcomes	Blood Loss > 375ml (median)	Fluid Intake > 3400ml (median)	Surgery Time > 5 hours (median)
ICU Stay ≥ 2 days	5.1 (0.1273)	1.8 (0.2296)	2.5 (0.0853)
Hospital Stay ≥ 7 days	2.3 (0.0008)	2.1 (0.0027)	2.7 (0.0003)
Cardiac Events	5.1 (0.2865)	5.1 (0.2865)	5.1 (0.2865)
Neurological Complications	9.2 (0.1317)	9.2 (0.1317)	9.2 (0.1317)
Postoperative Oxygen inhalation	2.6 (0.0392)	1.0 (0.9513)	2.0 (0.1076)
Respiratory Support (if needed)	2.5 (0.3903)	2.5 (0.1334)	5.0 (0.0134)
Acute Kidney Injury	5.1 (0.1273)	0.7 (0.7192)	5.1 (0.1273)

**Table 3:** Odds ratio of outcomes to intraoperative factors with p-values. p-value significant: <0.05 ICU: Intensive care unit

Outcomes	Blood Loss > 375ml(median)	Fluid Intake > 3400ml(median)	Surgery Time > 5 hours(median)
ICU Stay ≥ 2 days	2.5 (0.1480)	2.1 (0.2244)	3.1 (0.0778)
Hospital Stay ≥ 7 days	5.9 (0.0601)	1.0 (0.9743)	7.6 (0.0001)
Cardiac Events	5.4 (0.2814)	5.4 (0.2814)	5.4 (0.2814)
Neurological Complications	10.2 (0.1226)	10.2 (0.1226)	10.2 (0.1226)
Post-Op Oxygen inhalation	3.5 (0.0327)	1.0 (0.9513)	2.5 (0.1006)
Respiratory Support (if needed)	2.1 (0.3874)	2.1 (0.3874)	5.7 (0.0126)
Acute Kidney Injury	5.7 (0.1190)	1.0 (0.9743)	5.7 (0.1190)

**Figure 2:**
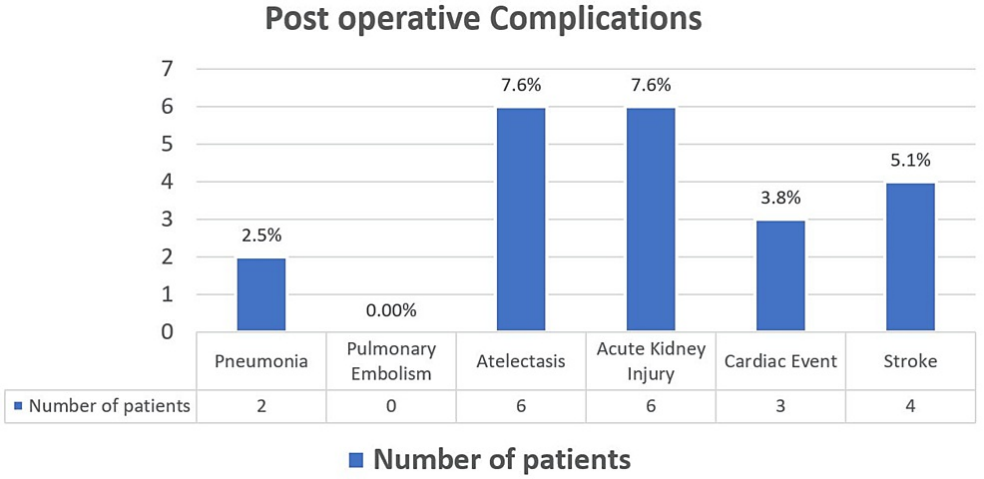
Postoperative complications.

Only 8.8% (n=7) of the patients were transfused intraoperatively, of whom 3.8% (n=3) required multiple transfusions (Figure 2). The calculated morbidity rate was 27.8% (n=22). The six-month mortality rate was 6.3% (n=5). Only one patient required re-exploration. However, 11.3% (n=9) of patients were lost to follow-up at six months.

## Discussion

Liver resection is the primary treatment for hepatobiliary cancer. The perioperative outcomes of the patients depend on several factors. In our study, 86.1% (n=68) of the patients who underwent major hepatic surgery were older than 40 years, predominantly hypertensive (36.7%, n=29), diabetic (29.1%, n=23), and smokers (34.2 %, n = 26). Similar results were reported by Melloul et al. [6], who stated that most of the patients had hypertension, followed by diabetes and coronary artery disease. In one large trial [13], smoking was associated with 47-86% increased risk of hepatic cancer and alcohol 68-87% increased risk of hepatic cancer. In our country, alcohol consumption is lower than that in the Western world, which is why smoking is a superior risk factor, as per the results of this study.

Although there were multiple factors and all were interlinked, some values of the relative risk and odds ratio stand out to relate the increased incidence of bleeding with postoperative oxygen requirement, long duration of surgery with hospital stay of > 7 days, and respiratory support requirement. The association between postoperative respiratory complications and longer surgical times has also been documented [14]. Similarly, it has been observed in a retrospective trial that prolonged operative time is associated with longer hospital stays [15].

The prolonged surgery time was found to be due to extensive retroperitoneal disease, extrahepatic Roux-en-Y biliary reconstruction, or teaching to the trainees. Other confounding factors for prolonged hospital stay included postoperative pneumonia, biliary leak, and liver decompensation. The most common cause of extensive blood loss was liver cirrhosis, which led to the increased use of Pringle’s maneuver (a surgical maneuver used to interrupt the blood flow through the hepatic artery and portal vein to help control bleeding from the liver) and ultimately led to increased oxygen requirement in the postoperative period.

No direct relationship was observed between blood transfusions and oxygen inhalation. No significant data were available to support the association between oxygen requirement and increased fluid intake. There are no established guidelines regarding the amount of fluid that should be administered. There are many questions regarding the fluid type to be given, the liberal versus restrictive approach, and the use of blood products. All the literature points toward goal-directed therapy, where the physician decides on fluid administration based on the patient's condition, ongoing losses, urine output, blood loss, and type of surgery [16]. The same approach was adopted by most physicians in our study.

Other outcomes could not be directly linked to any single factor. Preoperative albumin and lactate levels alone were not directly associated with postoperative morbidity or mortality. Rather, it would be better to say that a collection of multiple factors, including comorbid conditions, disease status, surgical complications, and perioperative management of the patient together affected the outcome of the patient. The study was limited by the data available from the HIS. If any information was not documented in detail, it was not retrieved. No control group was used to compare results.

## Conclusions

In conclusion, patients in our cohort who had a prolonged duration of surgery and a large volume of blood loss were associated with prolonged hospital admission (>7 days) and required oxygen inhalation and respiratory support postoperatively. Prospective trials aiming to compare the above-mentioned findings are required to confirm the association. Also, other factors such as the operator (anesthetist and surgeon) can be fixed to get more precise results.
